# Abdominal, thoracic, and cardiac point-of-care ultrasound skills following an in-person hands-on training course for early-track emergency clinicians

**DOI:** 10.3389/fvets.2025.1520004

**Published:** 2025-07-08

**Authors:** Julien Guillaumin, Amanda Cavanagh, Jaime Rechy, Morgan Callahan, Rita Hanel

**Affiliations:** ^1^Department of Clinical Sciences, College of Veterinary Medicine and Biomedical Sciences, Colorado State University, Fort Collins, CO, United States; ^2^VEG-ER for Pets, White Plains, NY, United States

**Keywords:** POCUS, focused ultrasound, competency, learning, assessment, performance decay

## Abstract

**Introduction:**

This study was designed to assess baseline compared to three months procedural skills performing abdominal, thoracic, and cardiac point-of-care ultrasound (POCUS) after POCUS training.

**Methods:**

A POCUS training was designed as a 3-hour online course, followed by a 2-day in-person course consisting of 3.5 hours of case-based lectures and 4 hours of hands-on laboratory on anesthetized dogs each day. In-person procedural assessment was performed using an anesthetized dog and consisted of identifying 22 anatomical structures in 6 minutes. The assessment was performed pre-course and repeated three months post-course in an identical environment.

**Results:**

Fifty-six veterinarians from the Veterinary Emergency Group New ER Doctor program were enrolled. Participants identified an overall 7.8 ± 2.6 structures in the pre-course assessment, compared to 13.8 ± 5.9 in the post-course assessment (*p* < 0.0001). For abdominal POCUS, participants identified 5.9 ± 1.9 structures out of 12 in the pre-course and 9.0±1.5 in the post-course assessment (*p* < 0.0001). For thoracic POCUS, participants identified 1.7 ± 1.2 structures out of 4 in the pre-course and 3.4±0.7 in the post-course assessment (*p* < 0.0001). For cardiac POCUS, participants identified 0.07 ± 0.3 structures out of 6 pre and 1.5±1.6 post-course assessment (*p* < 0.0001). There was no impact of pre-course tested variables on the pre-course score. Survey-based course satisfaction was 100%.

**Discussion:**

The Veterinary Emergency Group New ER Doctor POCUS course improved participants’ ability to correctly identify anatomical structures on POCUS when assessed three months after the course.

## 1 Introduction

Initially designed by trauma surgeons to assess trauma patients bedside, point-of-care ultrasound (POCUS) has evolved to be ubiquitous in emergency medicine in people (1, 2). POCUS is defined as ultrasonography at the patient’s bedside that is performed in real-time by a clinician caring for the patient (3). In contrast to traditional comprehensive ultrasound examinations that may involve multiple providers and steps, diagnostic POCUS examinations involve the same clinician determining the need for a focused examination, acquiring and interpreting the images, and incorporating the findings into the immediate management of the patient. POCUS use has increased in emergency medicine, critical care medicine, and internal medicine over the past two decades (4).

In veterinary emergency and critical care medicine, POCUS is similarly an important part of practice and training (1, 2, 5–7). The first report of abdominal POCUS (A-POCUS) was published in 2004 in trauma dogs and the first report of thoracic POCUS (T-POCUS) in 2008 (8, 9). A focused cardiac POCUS (C-POCUS) procedure was also recently described (10). However, incorporation of POCUS training in veterinary medical education is lacking, especially compared to medical schools. Currently, almost 73% of medical schools include POCUS in their preclinical courses as well as clinical education, and a specific C-POCUS curriculum for medical students has been created (11–14). This number is presumably much lower in veterinary medicine. In one survey, veterinarians stated that not having an ultrasound machine in their practice and lack of training or education were the most common reasons for not performing ultrasound exams (6). Recent studies have investigated the feasibility of self-driven POCUS learning, in-person and online video POCUS instructions as well as an hybrid online, in-person didactic training and hands-on training (15–23).

Existing studies vary in scope, target audience and design. The majority of studies investigate veterinary students (15–18, 20, 21). Only two studies investigated canine primary practitioners in the United Kingdom or Australia (21, 23). Most studies focused on a specific organ, such as kidney or heart, one investigated a four quadrant A-POCUS, and only one investigated a more global POCUS approach with A-POCUS, T-POCUS and C-POCUS (16, 20, 22, 23). Published study outcomes also vary, including image quality assessed by an expert (15, 16, 20, 22), comparison to images acquired by board certified specialists (19, 21), and satisfaction survey (18, 23).

In people, studies have demonstrated the effectiveness of POCUS training courses on knowledge and skills acquisition by different learner groups. However, these courses have varied in duration, content, delivery, and target audience (4, 24–26). Similarly, there was heterogeneity in outcome assessment of POCUS competencies. Ideally, a course teaching POCUS should lead to an improvement in ultrasound competency, would lead to students’ satisfaction, and students should retain their skills over time.

The aim of our study was to assess the effectiveness of a 2-day POCUS course targeting novice learners in an immersive emergency doctor training program. Our primary objective was to compare baseline skill to a 3-month post-course evaluation assessing learners’ ability to correctly identify specific anatomical locations in A-POCUS, T-POCUS, and C-POCUS. We hypothesized that novice learners will improve their POCUS skills globally 3-months after the POCUS course compared to baseline. Our secondary objectives were to determine if any predictors of higher skill levels existed and to assess course feedback, including participants’ satisfaction. We hypothesized there would be some predictor(s) of higher skill levels, and that participants would be satisfied with the course and their improved skill level.

## 2 Materials and methods

### 2.1 Course design

The POCUS course was designed by Veterinary Emergency Group (VEG) as a 3-prong hybrid approach. First, a 3-h online module consisting of 1-h lectures recorded by experts in their respective fields of A-POCUS, T-POCUS, and C-POCUS was hosted in a learning management system and was accessible remotely at any time by the participants. The lectures included terminology commonly used in POCUS, using terms referring to ultrasound (US) probe location or anatomic site, without any references to trademarked protocols. The lecture content showed participants ways to acquire images of various anatomical structures, including those listed on Table 1, and instructed participants to follow a systemic approach to POCUS of anatomical regions such as starting at the subxiphoid view and making a clockwise or counterclockwise approach around the abdomen, the check-mark sign for T-POCUS and a mushroom view start for C-POCUS (10, 27).

**Table 1:** Pre and post-POCUS course ability to correctly identify an anatomical local by junior emergency veterinarians (*n* = 56).

Anatomical location number	Anatomical location	Pre-course assessment performance (% unless specified)	Post-course assessment performance (% unless specified)
Abdominal point of care ultrasound (A-POCUS)			
1	Liver	82	91
2	Gallbladder	70	93^*^
3	Heart through the SX view	48	86^*^
4	Caudal vena cava	2	20^*^
5	Stomach	32	71^*^
6	Right kidney	27	75^*^
7	Bladder	96	98
8	Bladder neck	57	79^*^
9	Aortic trifurcation	0	43^**^
10	Left kidney	84	96^*^
11	Spleen	80	95^*^
12	Splenic hilus	14	48^*^
			
	Systemic approach	18	68^*^
	Time spent in A-POCUS (sec) ^#^	241.4 ± 58.3	184.9 ± 35.3^*^
Thoracic point of care ultrasound (T-POCUS)			
13	A-line	41	85^*^
14	Bone-Air in Transverse sign	34	90^*^
15	Glide sign	77	90^*^
16	Seashore sign	23	56^*^
			
	Systemic approach	18	54^*^
	Time spent in T-POCUS (sec) ^#^	65.6 ± 37.9	67.5 (30–180)
			
Cardiac point of care ultrasound (C-POCUS)			
17	Right parasternal short-axis ventricular view (i.e., Mushroom view)	5	49^*^
18	Right parasternal short-axis heart base view (i.e., Left atrium to Aorta view)	0	24^*^
19	Main pulmonary artery	0	7^*^
20	Right parasternal long-axis view identifying LA, right atrium, left ventricle and right ventricle view (i.e., Four chambers view)	2	22^*^
21	Right parasternal long-axis view identifying LA, Ao, right atrium, left ventricle and right ventricle view (i.e., Five chambers view)	0	10^*^
22	Mitral valve in a right parasternal short or long-axis view	0	27^*^
			
	Systemic approach	0	5
	Time spent in C-POCUS (sec) ^#^	53.9 ± 44.6	101.4 ± 38.3^*^

SX, sub-xyphoid; sec, seconds. *p < 0.05. #: represents the mean ± standard deviation or median (minimum-maximum) of the individual participant’s times.

Second, participants were enrolled in a 2-day in-person course consisting of lectures and hands-on training. A total of 3.5 h per day of case-based lectures covering the various aspects of POCUS use in emergency practice (7 h total) was delivered in-person by content experts. The primary learning objective for the lectures was the incorporation of POCUS in case workup and management (e.g., canine spontaneous hemoabdomen for A-POCUS, feline pyothorax for T-POCUS, and feline congestive heart failure for C-POCUS). The cases assumed sufficient knowledge of POCUS techniques based on the supplied pre-course work. Participants also had a 4-h per day, in-person, hands-on POCUS training on anesthetized, purpose-bred, ethically sourced Beagles (8 h total). Due to the number of participants, the hands-on training portion included two groups of participants. For each group of participants, the hands-on training portion had 8-stations divided into two identical groups of four stations.

There were a total of four training stations on each day. On Day 1, there was one A-POCUS in left lateral recumbency, one T-POCUS in sternal recumbency, one C-POCUS in left lateral recumbency and one free scan station in right lateral recumbency where participants could choose any of the three POCUS techniques or a mix of the three. On Day 2, there was one A-POCUS in left lateral recumbency, one T-POCUS in sternal recumbency, and two C-POCUS stations in left lateral and right lateral recumbency. The participants were not allowed to move the dog into a different position unless deemed necessary by the instructor. For C-POCUS views and the right kidney view in right lateral recumbency, students were instructed to slide the probe underneath the dog. Each station had approximately four participants per station, and each group of four participants rotated every 60 min per station. Therefore, each participant had an approximate time of 15 min at four different stations over 2 days, for a total of 120 min of actual ultrasound scanning time.

An instructor was assigned to each of the stations. Instruction was at the discretion of the specific instructor and stations. Each instructor had access and was asked to review in advance the same 3-h online module available to the students, including specific systemic approaches. Most of the instructors were familiar with the hands-on training and had taught it before. No specific effort was made by the instructors to review the list of anatomical structures that were used during the assessment phase, although individual participants could ask for clarification and help, especially during the free scan station. Instructors were all American board-certified in either cardiology or emergency and critical care, emergency and critical care residents, or experienced academic emergency room doctors with POCUS teaching experience. The course was gamified using rapid rotation between participant and stations (15 min), engaged and present instructors, an individualized bingo sheet that highlighted the 22 anatomical locations assessed during the study, and the occasional use of a spinning wheel with specific anatomical locations to identify in a certain time frame (28).

The course design adopted for the study was based on previous experience by the study team in POCUS training for VEG NERDS for the past 2 years, or approximately six training sessions with similar, but not identical design. Many of the decisions made for the current study design were made due to those previous experiences.

### 2.2 Participant’s inclusion criteria

Participants for the study were enrolled in Veterinary Emergency Group’s New ER Doctor (NERD) program. NERDs were defined as graduates veterinarians without experience in emergency medicine and were mostly veterinary school graduated within the past 6 months. Prerequisites for the POCUS course included completing the online lectures, answering five quiz questions for each hour of lecture, and having a passing grade of 12 correct answers out of 15 questions.

Participants were excluded if they: were not a VEG NERD, did not complete the required online course, did not have a passing grade for the online course, did not perform both assessments (pre-course and post-course), or were unable to perform a POCUS due to injury.

### 2.3 Study design

Identical in-person pre-course and post-course hands-on assessments were performed. The pre-course assessment was performed on the first morning of the course, as participants were pulled out of lectures or breaks for approximately 10–15 min. The post-course assessment was done in a similar fashion, in-person, 3 months post-course. The same dog was used for each participant for pre-course and post-course assessments.

Participants signed an informed consent prior to the course and were again briefed on the study in-person, the morning of the pre-course assessment, and were provided with the list of anatomical locations (Table 1). They were again briefed on the study in-person the morning of the post-course assessment. Each participant was pre-assigned a time slot (similar for the pre-course and the post-course assessment).

For each of the four anesthesia events, the dog was pre-medicated with butorphanol (0.2–0.4 mg/kg IM, *n* = 4) and dexmedetomidine (5 mcg/kg IM, *n* = 2), or atropine (0.02 mg/kg IM, *n* = 2). After an intravenous catheter was placed, anesthesia induction was performed using ketamine (4 mg/kg IV, *n* = 4) and midazolam (0.2 mg/kg IV, *n* = 4). Maropitant (1 mg/kg SQ) was administered during two of the four anesthesia events. The dog was maintained under light anesthesia with inhaled isoflurane after orotracheal intubation and was breathing spontaneously. Atropine (0.02 mg/kg IV or IM) was used at the anesthesia technician discretion to maintain heart rate above 100 bpm. Dopamine (5–7 mcg/kg/min) was used at the anesthesia technician discretion to maintain blood pressure above 65 mm Hg.

**Figure 1:**
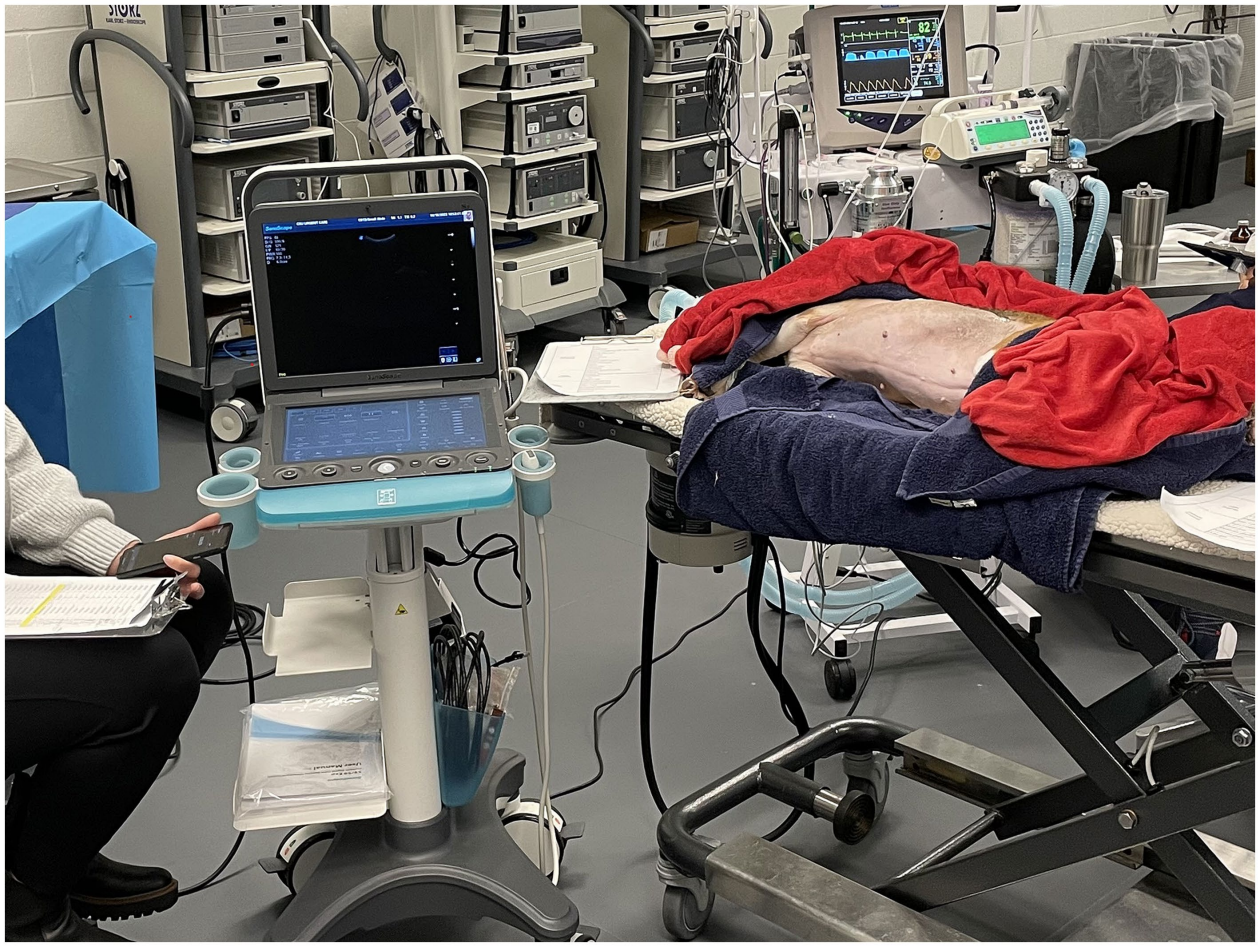
Point-of-care ultrasound (POCUS) course assessment set-up. An ethically-sourced, purpose-bred Beagle was anesthetized, monitored (anesthesia technician visible on the right), and placed in right lateral recumbency. The participant (center) would point on the screen the anatomical location and the assessor (left) would move the ultrasound machine’s cursor on the anatomical location pointed by the participant. Three videos-captures were performed: a bird-view of the dog, a recording of the ultrasound machine input displaying the arrow/cursor (video graphics array cable) and a video recording of the screen displaying hand position.

The anesthetized dog was placed in a right lateral recumbency, and the abdomen and left thorax were clipped (Figure 1). A lateral recumbency was chosen due to the anesthesia limiting the ability to maintain sternal recumbency without external holding device which would limit access to the chest of the dog. A right lateral recumbency was chosen for practical reasons, including the dog’s abdomen facing the participant’s position at the left of the examination table (Figure 1), as well as the authors’ experience with shifting of the cardiac axis secondary to atelectasis during the 4 h anesthesia time needed to assess all participants, therefore making C-POCUS image acquisition variable over the course of the assessment. All participants stood left to the dog and performed the ultrasound exam with their right hand. Participants were not allowed to move the dog in a different recumbency side and therefore had to slide their hand underneath the dog in order to acquire C-POCUS images as well as right kidney. In the author’s experience, this is performed very easily with the small size dog used in this study.

The participants were given 6 min to perform an A-POCUS, T-POCUS, and C-POCUS and identify as many of the 22 anatomical locations as possible (Table 1). Participants were not allowed to move the dog into a different position. Ultrasound gel was used although alcohol was withheld due to concerns regarding hypothermia. A study support person not involved in the course was present to answer any questions regarding the ultrasound machine^1^ (i.e., knobology), switch the machine to a cardiac mode if/when instructed by the participant, and had a physical copy of the list of anatomical locations that the participant could also consult (Table 1). They were not allowed to answer any questions related to the study or the anatomical structure seen on screen. Their default answer was “*do your best*,” and they wore a face mask to avoid showing facial expressions. The total study time was 6 min per participant, and the study support person provided a 2 min and four-minute countdown and dismissed the participant after 6 min.

Each participant was equipped with a lapel microphone and instructed to point with their finger on the screen to the specific anatomical location and verbalize the site/sign they identified. The study support person would move the US cursor to the area underneath the participant’s finger for redundant video recording purposes. Three video-captures were performed: a bird’s-eye view of the dog, a recording of the ultrasound machine input displaying the arrow/cursor (video graphics array cable), and a video recording of the screen displaying hand position (Figure 1).

### 2.4 Data collected and outcome measured

Prior to the study, VEG collected data from the participants, including age, gender/gender identity, dominant hand, veterinary school attended, if the participant received POCUS training during veterinary school, graduation year, and a self-rated POCUS competency using a scale of 0 (i.e., novice) to 5 (i.e., expert). VEG also collected a post-course satisfaction survey that included a second self-rated POCUS competency using the aforementioned scale. The post-course satisfaction survey was submitted at the end of the training course and included two questions, one with a binary answer (i.e., Yes/No) regarding perception of the course benefit for skills improvement, and one using the aforementioned 5-points scale for self-rated POCUS competency.

All data from the 3 videos-captures from both sessions were acquired, then anonymized and labeled by a person not involved in the study design or the course to anonymize the participant and the assessment session (i.e., pre or post). That person was provided with a randomized table and re-labeled each video from 1 to 112. Those randomized, anonymized videos were provided for analysis to one of the authors (AC) not involved in the study design or the hands-on lab portion. One point was granted if the participant correctly identified and verbalized the anatomical structure. Correct identification was defined by the image being able appropriate enough to be shown in a lecture or a textbook used by a board-certified emergency and critical care specialist trained in POCUS.

For the purpose of our study, we defined an individual organ (such as the right kidney), an anatomical artifact (such as the bone-air in transverse artifact in T-POCUS) or a specific view (such as a “mushroom view”) listed in Table 1 as an “anatomical structure,” and we used the term anatomical regions to describe A-POCUS, T-POCUS or C-POCUS. The total number of anatomical structures identified, as well as the total number of anatomical structures by anatomical regions were recorded. Identification of each individual anatomical structure was also recorded (Table 1). If a systematic approach was followed, defined as starting at the subxiphoid view and making a clockwise or counterclockwise approach around the abdomen, the check-mark sign for T-POCUS and a mushroom view start for C-POCUS, it was recorded as a binary outcome (i.e., Yes/No) (10, 27). The time spent by each participant in each region was also recorded.

The study was approved by the Colorado State University Institutional Animal Care and Use Committee (#KP 1258) as well as Institutional Review Board (#KP 5927). Written informed consent was obtained from all study subjects before participation.

### 2.5 Statistical analysis

Preliminary data (not shown) with a group of participants (*n* = 77) not enrolled in the study, but enrolled in an earlier similar, but not identical program, determined they were able to identify 78 ± 15% of anatomical structures that were over 90% similar to the one used in the current study in 5 min 3 months after the POCUS training. Assuming the participant will improve from a baseline score of 60% to a score of 75% with a standard deviation of 15%, 10 students were needed to achieve significance for our primary outcome using a paired samples t-test. A recruitment target of 50 paired assessments was due to the VEG NERDS program coming as cohort of 60 or more doctors, and to mitigate against smaller observed differences.

D’Agostino-Pearson test was used to assess normal distribution. Data were presented in mean ± standard deviation, or median (minimum-maximum), as appropriate. Paired *t*-tests, or Wilcoxon paired tests, as appropriate, were performed to assess differences between numbers of structures identified between the pre and the post-course assessment for the total numbers of structures identified, as well as number of structures identified for each of the regions of A-POCUS, T-POCUS, and C-POCUS.

Factors influencing pre-course scores, and post-course improvement were examined via stepwise multiple regression. Age, pre-course quiz average, dominant hand, veterinary school rank according to the United States news 2023 report,^2^ having a POCUS course or hands-on laboratory during veterinary school training (self-disclosed) and pre-course self-rating competency were included as independent variables. The dependent variables tested were pre-course scores and the post-course improvement above the average post-course assessment + one standard deviation. Independent variables were removed if *p* > 0.1. Statistical significance was set at *p* < 0.05. All statistical analyses were performed using commercial software.^3^

## 3 Results

### 3.1 Dogs

Purpose-bred spayed female Beagles between 1 and 2 years of age with a mean weight of 12.0 ± 1.8 kg were ethically sourced. Dogs were acclimated for a minimum of 1 week prior to baseline health assessments. Daily enrichment was provided, and group housing was performed, if possible, according to Colorado State University Laboratory Animal Resources protocols. Free access to water was allowed and a commercial dry kibble was fed twice daily. Food was withheld for 12 h prior to anesthesia. An anesthetized Beagle was used both for the assessment and the hands-on lab (#KP 1258). The same dog was used for both the pre-course assessment and the post-course assessment. All dogs used in the study were ultimately adopted.

### 3.2 Study participants demographics

Sixty-seven participants took the pre-requisites and hands-on course. All participants attended the online lectures and received a 75% or above pass rate on the quiz. Eleven participants were excluded as they graduated veterinary over 6 months prior to the course (*n* = 7), did not perform both pre and post course assessment due to sickness (*n* = 2), a family emergency (*n* = 1) or leaving the NERD program (*n* = 1). Therefore, 56 participants were included in the study for a total of 112 videos. The post-course assessment took place an average of 81 ± 23 days after the pre-course. Median age was 26.0 (25–55) years old. The majority of participants identified as female (n = 54, 96.4%) and two participants identified as male (3.6%). The majority of participants were right-handed (n = 48, 85.7%), while the remaining were left-handed (*n* = 8). All participants graduated in 2022 from the following veterinary schools: University of Florida (*n* = 6), Oklahoma State University (*n* = 5), University of Georgia (*n* = 5), Auburn University (*n* = 4), Tufts University (*n* = 3), Texas A&M University (*n* = 3), St. George’s University (*n* = 3), Cornell University (*n* = 3), Colorado State University (*n* = 2), The Ohio State University (*n* = 2), Louisiana State University (*n* = 2), University of Missouri (*n* = 2), University of Illinois (*n* = 2), University College of Dublin (Ireland) (*n* = 2), and 1 of each of the following universities: Kansas State University, Lincoln Memorial University, Mississippi State University, North Carolina State University, Ross University, University of Tennessee, University of Minnesota, University of Pennsylvania, University of Wisconsin, Virginia Maryland University, Western University and the Royal Veterinary College (England). Approximately half of the participants (*n* = 31, 55.3%) did not recall having a POCUS lecture or hands-on lab during veterinary school while 44.7% recalled having one (*n* = 25). The mean self-rated POCUS competency was 1.8 ± 1.0 on a 5-point scale, 0 being an inexperienced user and 5 being a self-rated expert.

### 3.3 Primary objective

When A-POCUS, T-POCUS, and C-POCUS were combined, participants identified 7.8 ± 2.6 out of 22 anatomical structures (35%) in the pre-course assessment, compared to 13.8 ± 5.9 out of 22 anatomical structures (63%) in the post-course assessment. The difference in anatomical structures identified was statistically significant between pre- and post-course assessment (*p* < 0.0001).

The improvement between pre- and post-course assessment was also statistically significant for each of the anatomical regions. For A-POCUS, participants identified 5.9 ± 1.9 out of 12 abdominal anatomical structures (49%) in the pre-course and 9.0 ± 1.5 out of 12 (75%) in the post-course assessment (*p* < 0.0001). For T-POCUS, participants identified 1.7 ± 1.2 out of four thoracic anatomical structures (42%) in the pre-course and 3.4 ± 0.7 out of four (85%) in the post-course assessment (*p* < 0.0001). For C-POCUS, participants identified 0.07 ± 0.3 out of six cardiac anatomical structures (1%) pre- and 1.5 ± 1.6 out of 6 (25%) post-course (*p* < 0.0001).

Each individual structure on A-POCUS, T-POCUS, and C-POCUS was correctly identified on the pre-course assessment in 0–96%, 23–77%, and 0–5%, respectively, depending on the individual structure (Table 1). Most participants did not follow a systematic approach to POCUS in the pre-course assessment (Table 1). Time spent on A-POCUS, T-POCUS, and C-POCUS was 4.0, 1.1, and 0.9 min, respectively, in the pre-course assessment (Table 1). Anatomical structures that were difficult to identify in the pre-course assessment, defined as a score less than 80%, had a significant increase identification in the post-course assessment (Table 1). The range of correctly identifying an anatomical location on the post-course assessment on A-POCUS, T-POCUS, and C-POCUS was 20–96%, 56–90% and 7–49%, respectively (Table 1). Most participants did follow a systematic approach to A-POCUS and T-POCUS, but not C-POCUS in the post-course assessment (Table 1). Time spent on A-POCUS decreased significantly, and time spent on C-POCUS increased significantly in the post-course assessment compared to the pre-course assessment (Table 1).

### 3.4 Secondary objectives

**Figure 2:**
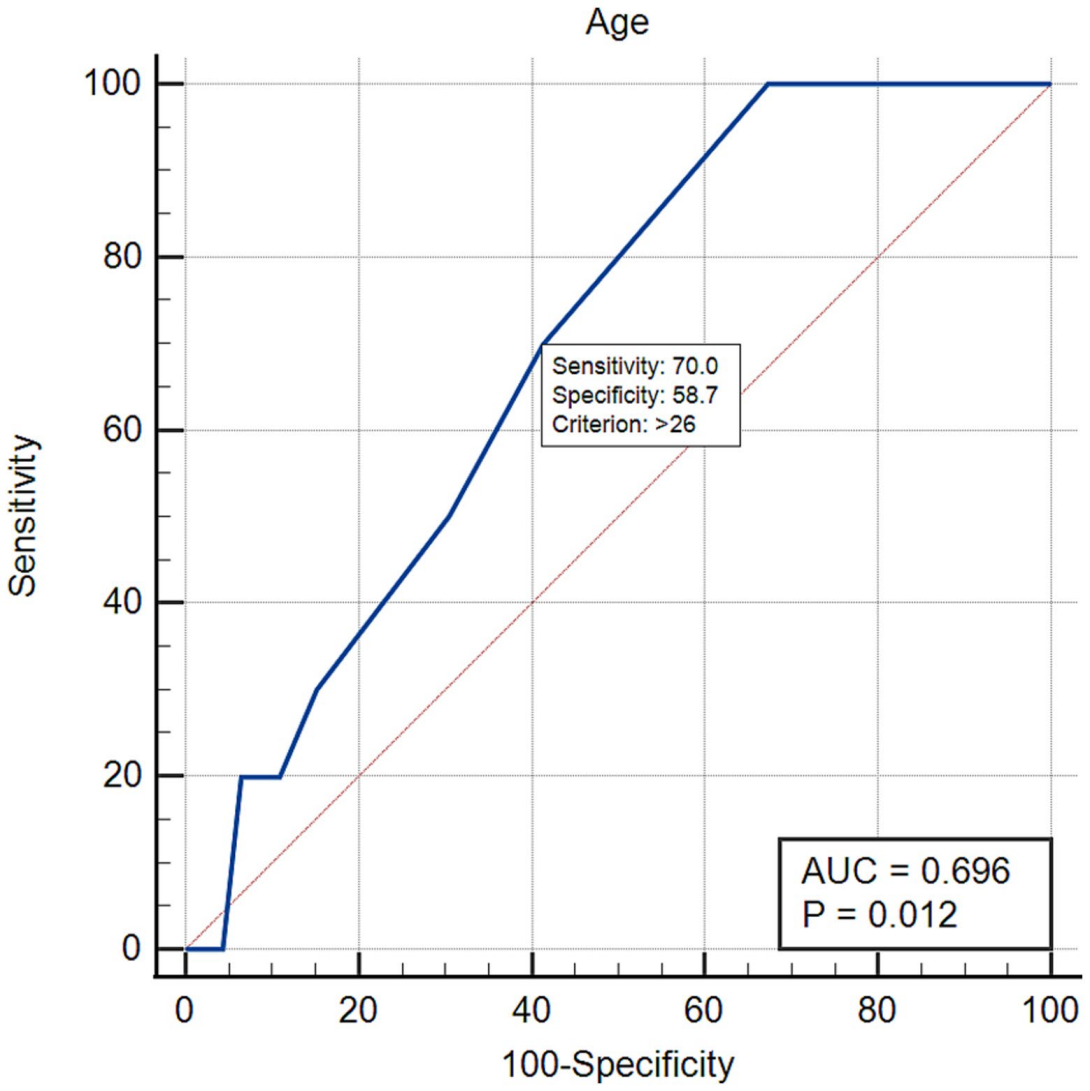
Receiving operating characteristics curve for the variable “age,” an independent predictor of increased performance in the post-course assessment, defined as average post-course improvement + one standard deviation.

None of the tested variables (i.e., age, pre-course quiz average, dominant hand, vet school rank, having a POCUS course or lab during veterinary school training and self-rating competency) impacted the pre-course score. The only tested variables impacting the post-course improvement was age, with older students performing better, with an estimated odds-ratio of 1.84 (95% CI 1.06–3.19, *p* = 0.029), meaning older students were 84% more likely to perform better. Receiver operating characteristics curve showed that a cut-off of 26 years was associated with higher likelihood of a post-test score above our threshold criterion, with a sensitivity of 70.0% and a specificity of 58.7% (AUC 0.696, *p* = 0.012) (Figure 2).

Twenty-four post-course surveys were received, 3 being duplicates and one could not be anonymized due to technical issues. Therefore, 20 individual surveys were analyzed (35.7% of participants). To the question “*Do you believe that this POCUS course was beneficial to improve your skill set?*,” 100% (*n* = 20) of the participants answered “*Yes*.” For the participants who answered the survey, the mean self-assessment score went from 1.9 (1.2) to 3.2 (0.7) (*p* < 0.0001).

## 4 Discussion

Our study found that our POCUS course designed for early career emergency clinicians resulted in a statistically significant improvement in correctly identifying anatomical structures 3 months after the course. The course satisfaction was excellent, and the participants reported an improvement in their self-assessment of POCUS skills. However, the post-course overall result of 63% of correctly identified anatomical structures should be considered a moderate proficiency. This is especially true for C-POCUS, where participants were only able to identify on average 25% of the anatomical structures. A-POCUS and T-POCUS’s retention of clinical skills were better, with 75 and 85% of anatomical structures correctly identified, respectively.

This was the first time global POCUS clinical skills training in small animal emergency veterinarians was investigated. Most available studies on veterinary POCUS training involved veterinary students, compared to recent graduates in our study (15–20). Less than 50% of our course participants recalled having some POCUS training, which should be contrasted to the 78% of students recalling having some didactic or hands-on training in a study from one US veterinary school (19). This can be due to the heterogeneity of veterinary schools the participants in our study come from.

Participants in our study self-reported an average of 2 out of 5 POCUS competency in our study, corresponding to a low to mild level of proficiency. Although not directly comparable, it seems a higher self-reported proficiency compared to American veterinary students reporting a median comfort level consistent with “*I have no prior training and have not seen them [POCUS study] done before*,” or less than 15% Australian practitioners being confident or very confident in various aspects of POCUS (16, 23).

Our primary outcome measurement was A-POCUS, T-POCUS and C-POCUS skills in dogs, with 22 anatomical structures studied and images reviewed by a board-certified emergency and critical care specialist. In A-POCUS, our 12 anatomical structures has to be contrasted with other small animal studies investigating a four quadrant A-POCUS (16), or appropriate identification and orientation on a single kidney view (20). In C-POCUS, our six anatomical structures has to be contrasted with two to four cardiac ultrasound views (19, 21). In previous studies, no clear explanation of how the landmark was identified was provided or reviewed (16), or images were reviewed by cardiologists (19, 21), or radiologists (20).

All other studies were designed for large animals (15, 17, 22), or had participant satisfaction and self-assessment as an outcome (18, 23). Participant satisfaction and self-assessment was also an outcome in the present study. Participant satisfaction of 100% was higher to the published ones of 83–94%, and their self-assessment of skills increased, similar to a previously published study (17, 18, 23).

Currently, there is not a universal-recognized standardized way to perform an A-POCUS, T-POCUS, or C-POCUS, known in people as extended FAST (e-FAST) or FAST-Pleural ultrasound (FAST-PLUS) (29–31). At the time of writing this manuscript, an expert consensus on POCUS protocol is underway and therefore a more standardized list of anatomical sites to evaluate during POCUS examination may be available following the consensus. Another consensus, by The American College of Veterinary Radiology and the European College of Veterinary Diagnostic Imaging has been recently published their consensus on how to perform a full abdominal ultrasound, although it is not relevant to POCUS evaluation (32). For T-POCUS and C-POCUS, clinicians refer to veterinary textbooks for didactic training, online training and/or in-person training (27, 33, 34). Our study design elected to expand the number of POCUS anatomical locations compared to the original abdominal focused assessment with sonography for trauma (FAST), and our course design is relatively similar to one recently published (8, 23). Although arbitrary, the A-POCUS sites were chosen to include important landmarks for specific emergency situations, such as diagnosing a splenic torsion with the “arrow sign” at the splenic hilus, or an aortic thrombus in dogs and cats (35–37). The anatomical locations we used for T-POCUS and C-POCUS have been previously described (10, 33, 38).

Participants had 22 anatomic structures to identify in 6 min, or 16 s per landmark. Pre-course, time allotment was skewed toward A-POCUS, with an average allowance of 20 s per 12 landmarks (more than 16 s), versus C-POCUS, with an average allowance of 9 s per 6 landmarks (less than 16 s). In the pre-course, participants may have spent too much time looking for A-POCUS anatomical structures, leaving less time for the C-POCUS anatomical structures. In the 3-month follow up assessment, the speed of performing A-POCUS decreased by almost 1 min, allowing participants more time to focus on other parts of the POCUS exam. As a result, when the time spent in each area is divided by the number of anatomical locations to identify, all three areas were more evenly distributed with an average of 15 s, 18 s, and 17 s per landmark available for A-POCUS, T-POCUS, and C-POCUS, respectively. This is very close to 16 s per landmark mentioned earlier. This should be contrasted with the aforementioned study in veterinary students, in which students were allotted 6 min to identify the 4 original landmark sites of an abdominal FAST, providing an average allotment of 90 s per site, significantly more than the 16 s in our study (16). The amount of time allocated per site is appropriate for the level of education of the participants, 90 s for veterinary students, compared to 16 s for emergency veterinarians (16). Another study allotted 6 min to veterinary students and veterinarians of various level of training to acquire five standard right-parasternal long- and short-axis C-POCUS views (22). This correspond to 18 s per site, almost identical to the 17 s per site from our study’s participants. We conclude that the improved speed in A-POCUS post-course allowed emergency veterinarians to perform A-POCUS, T-POCUS and C-POCUS in a clinically relevant time.

After the POCUS course, participants showed significant improvement, and moderate retention of their ability to correctly identify A-POCUS sites. Eighty percent or more of participants were already able to correctly identify the urinary bladder, left kidney, liver, and spleen on the pre-course baseline assessment. The three least correctly identified A-POCUS sites in the pre-course test were the aortic trifurcation, caudal vena cava, and splenic hilus. After the POCUS course, all of the A-POCUS anatomical locations that scored less than 80% at baseline showed a significant increase in the learners’ ability to correctly identify it. Of interest, the liver, right kidney, urinary bladder, and left kidney were identified by 91, 75, 98 and 96% of participants, respectively. The participants in our study performed better compared to the veterinary students investigated in the aforementioned study, where the correct identification of these landmarks within 4 anatomical views were 50, 19, 64, and 22%, respectively (15). This difference could be related to the previously discussed pre-course POCUS comfort scores, the previously discussed level of training and education of participants, or could be due to the course design itself. Regarding the course design differences, in the veterinary students’ study, one group of students had a live-animal training session for up to 20 min with one-on-one instruction, and one group had an online tutorial, and found a significant difference between the two groups in the students’ ability to identify the subxiphoid view, but not for the other three A-POCUS views, with the one-on-one instruction performing better (15). In our study, participants had both online coursework and 120 min of one-on-one instruction for all participants.

The performance of T-POCUS had a more variable result. Overall, participants showed statistically significant improvement in and retention of their T-POCUS skills. Indeed, the overall average post-course scores were highest in the T-POCUS landmarks. The glide sign was the most likely to be identified in the pre- and post-course assessments, followed by the A-line in the pre-course and the distal acoustic shadow produced by the ribs in the post-course test. There are no published studies to compare from.

Cardiac POCUS had the lowest overall scores, with participants identifying 0.07 ± 0.3 structures pre and 1.5 ± 1.6 post. For C-POCUS, the right parasternal short-axis view at the left ventricular papillary muscles (mushroom view) and the right parasternal long-axis 4-chamber view were correctly identified in the pre-course assessment by only 5 and 2% of participants, respectively. Direct comparison of our results with published studies cannot be made, as this specific information is not available in the literature (19, 23). After the course, the time spent on C-POCUS increased, and each of the C-POCUS anatomical locations showed an increase in the participants’ ability to correctly identify it. However, the most identified anatomic location, the mushroom view, was correctly identified by less than 50% of the learners. Explanation for this finding could be a lack of time, a positioning issue with the dog placed on right lateral recumbency without an echocardiography table, or cardiac anatomical shift due to atelectasis. Interestingly, these findings in our study are comparable to a study performed in a university setting with 23 medical students and one first year physician assistant on motor and cognitive skill retention for novice POCUS learners (39). The authors found that there was a higher rate of decay at 4 weeks for cardiac images than pleural or vascular images and hypothesized that this was due to the higher complexity and difficulty of cardiac imaging.

There were no predictors for performance in the pre-course assessment. Regarding the post-course assessment, however, older students tended to perform better in the post-course assessment. However, the cut-off of 26 years-old identified was identical to the median age of participants. The median age of the participants makes sense, are most of them were young adult learners. It is possible that our finding of older students performing better was due to only a few older participants. Although older adults (i.e., 65 years-old) can be slower and have a lower level of accuracy compared to younger adults (i.e., 24 years-old), it has been shown that the ability to acquire knowledge is largely unaffected by cognitive aging (40). A study in people investigated the neurocognitive mechanisms most important in competence development in performing POCUS (41). In that study, only “relevant knowledge” (i.e., multiple-choice tests, knobology, image interpretation and basic anatomical knowledge) and “visuospatial ability” (i.e., visuospatial manipulation and visuospatial perception), but not “psychomotor ability” were identified as determinants of POCUS competence development (41). If POCUS skills are more related to relevant knowledge and visuospatial ability, and that relevant knowledge is not affected by cognitive aging, it makes sense that older adults may perform POCUS as well as younger adults.

The course satisfaction was excellent, and the participants reported an improvement in their self-assessment of POCUS skills. The post-course survey documenting this had a response rate of 36%, which is consistent with current survey literature (42). There is growing interest and research surrounding the impact of self-assessments, confidence, or self-efficacy, on the competence of a learner (43). Recent surveys showed that the vast majority of primary care veterinarians used POCUS in some form, with the majority of them performing the procedure weekly or daily (6, 7). Competency evaluation was limited in that study with the most common reason cited for limited POCUS use being lack of confidence in performing the procedure (6, 7). Courses such as the one in this study and others in people and veterinary medicine have documented increases in self-efficacy (confidence), and as such, may ultimately improve competence through enhancing confidence (16, 18, 19, 23, 44).

Assuming proficiency was achieved post-course, the amount of decay was significant. In people, there is a growing collection of research, including a meta-analysis, on how to ensure learning retention and prevent decay. This can be done using successive relearning or spaced retrieval of concepts, which involves offering opportunities to retrieve course content beyond the usual short window of time following the initial learning (45–47). This contrasts with the well-established practice of mass learning at a conference or course, such as in this study. Studies specifically looking at retention and decay involving POCUS in human health care recommend retraining of skills at a maximum of 8 weeks from initial training for cardiac and pleural ultrasound in one study, with significant decay noted within 1 month in a second study (39, 48). Similar findings have been documented in other areas of healthcare that rely heavily on psychomotor skills, such as surgery (49). Although it is reasonable to believe that some participants practiced POCUS examination during the 3-month period in between assessments, it was not structured, recorded or formatted into a method involving relearning (testing), so the impact, or lack thereof, on decay cannot be discerned. This represents an area of future research for our group, in which we endeavor to include a post-course assessment, along with successive relearning, in an effort to measure its impact on retention of POCUS skills.

### 4.1 Limitations

Our study has some limitations. Some of them have already been discussed, such as the unknown clinical relevance of older participants performing better or the time limitation to identify 22 anatomical structures in 6 min. This timeframe was chosen because it is not only clinically relevant, but consistent with other studies (16, 22).

We did not perform an immediate post-course assessment, which limits our ability to accurately document an immediate post-course level of proficiency, and therefore proficiency decay over time. This was mainly due to not having time with the participants immediately after the course, as well as finances. The authors were also comfortable to forgo the immediate post-assessment because they believe, based on previous experience teaching this course and performing unrecorded mock tests similar to the one performed in our study, that the majority of participants should be able to identify the 22 anatomic locations within 6 min at the end of the 2-day course. This belief was confirmed by a study showing 100% success in obtaining C-POCUS images in horses after a 1-day course, and another study in medical students showing 100% in obtaining T-POCUS and C-POCUS images after a 1-h didactic and 1-h hands-on training (22, 39).

Because of the need for a standardized setup due to camera placements, the use of the non-dominant hand for a small percentage of the participants could have swayed the results, although it did not appear to affect the pre-course or post-course proficiency. Although limited, existing evidence suggests that using the non-dominant hand for ultrasound image acquisition or guidance has minimal impact on performance (50–53).

Unlike other veterinary studies, our study did not define specific image quality criteria for correct anatomical identification. However, previous studies focused on much fewer structures (e.g., 1–4), while our study examined 22 (20, 22). Our decision not to include strict criteria was based on logistical constraints creating 22 strict criteria for three anatomical regions.

## 5 Conclusion

New ER doctors significantly improved their skills in A-POCUS, T-POCUS, and C-POCUS when assessed 3 months after their POCUS course, meaning the course appears to be effective at teaching global POCUS skills. They reached moderate proficiency in A-POCUS and T-POCUS, but not C-POCUS.

## Data Availability

The raw data supporting the conclusions of this article will be made available by the authors, without undue reservation.

## Footnotes

^1^Sonosite S9, Universal Inc., Bedford Hills, NY, United States.

^2^https://www.usnews.com/best-graduate-schools/top-health-schools/veterinarian-rankings, consulted 5/5/2023.

^3^MedCalc^®^ Statistical Software version 20.210. MedCalc Software Ltd., Ostend, Belgium.
